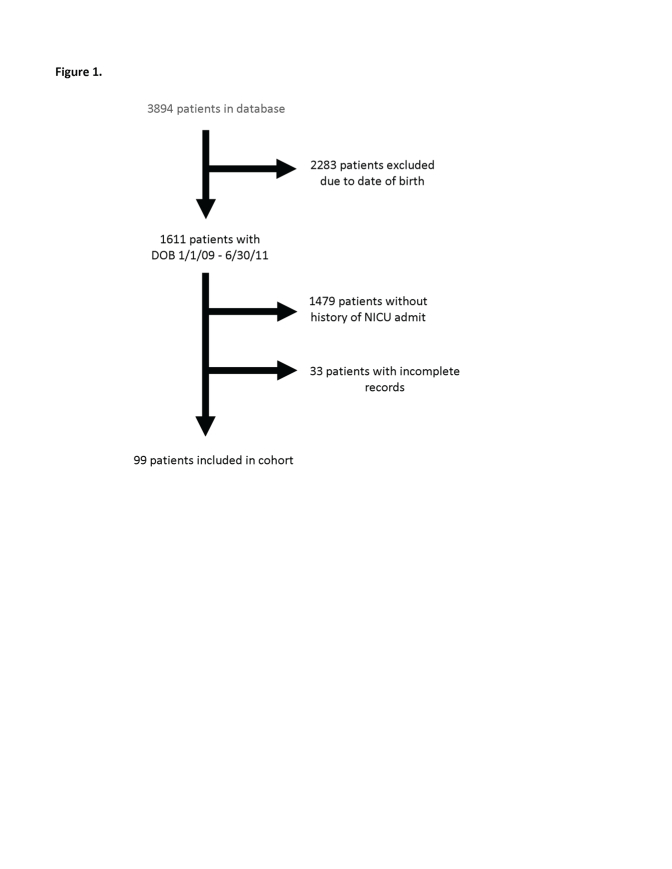# Clinical risk factors predicting likelihood of pathogenic genetic result in NICU patients

**DOI:** 10.1016/j.gimo.2026.104370

**Published:** 2026-01-30

**Authors:** Joshua L. Bonkowsky, Samuel B. Zoucha, Jenna Jensen, Jacob Wilkes, Rachel N. Palmquist, Mark Yandell, Martin Tristani-Firouzi

**Affiliations:** 1 Center for Personalized Medicine, Primary Children’s Hospital, Intermountain Healthcare, Salt Lake City, UT; 2 Division of Pediatric Neurology, Department of Pediatrics, University of Utah School of Medicine, Salt Lake City, UT; 3 Division of Neonatology, Department of Pediatrics, University of Utah School of Medicine, Salt Lake City, UT; 4 School of Medicine, University of Utah School of Medicine, Salt Lake City, UT; 5 Intermountain Healthcare, Salt Lake City, UT; 6 Department of Human Genetics, Utah Center for Genetic Discovery, Salt Lake City, UT; 7 Division of Pediatric Cardiology, University of Utah School of Medicine, Salt Lake City, UT

**Keywords:** Critical illness, Genetic testing, Genome, NICU, Risk factors

## Abstract

**Purpose:**

Children with neonatal intensive care unit (NICU) admission have higher rates of genetic disease, but it is unclear which patients should have genetic testing. Our goal was to identify clinical predictors associated with a pathogenic genetic result in NICU infants.

**Methods:**

This was a retrospective, population-based cohort analysis of infants born between January 1, 2009 and June 30, 2011 with a history of NICU admission and subsequent follow-up and genetic testing through 2021.

**Results:**

A total of 99 infants met inclusion criteria. In total, 64 (65%) patients had a negative genetic test result; 35 (35%) had a positive (pathogenic) result. Lower birthweight, younger gestational age, longer length of stay, or 2 or more indicators of severe illness were associated with a pathogenic result.

**Conclusion:**

Our work suggests that clinical predictors can be used to guide genetic testing in NICU infants. Use of these clinical risk factors could provide a diagnostic care pathway, potentially shortening the diagnostic odyssey and reducing potential morbidities.

## Introduction

Multiple studies have shown that children with a history of intensive care unit (ICU) admission have higher rates of genetic disease and that genetic disorders are a leading cause of ICU admission,^1^^,^^2^ but it is unclear which patients should have genetic testing. In the pediatric ICU population, genetic disorders are a cause of significant mortality and are associated with increased health care costs.^3^ The rate of positive (pathogenic test results) among children with an ICU admission is 20% to 30 %, and diagnosis affects care in approximately half of those with a genetic condition.^1^^,^^2^^,^^4^, ^5^, ^6^ Furthermore, 20% of all infant deaths are thought to be related to or have genetic disease as a direct cause.^7^^,^^8^ Indications for use of next-generation sequencing (NGS) in the clinical setting are uncertain.^9^ One study regarding the use of genome sequencing (GS) in the newborn intensive care unit (NICU) found that 45% of those diagnosed via GS had conditions not considered in the differential diagnosis.^10^ This underscores the ambiguity and limited understanding of the phenotypic presentations that the NICU population can display. Despite the lack of guidelines about which patients to test, there are many studies that perform genetic testing on critically ill children.^2^^,^^4^^,^^7^^,^^10^, ^11^, ^12^, ^13^, ^14^

Our goal was to determine whether clinical features, or markers of severity of illness, such as length of mechanical ventilation, inotrope administration, or antibiotic administration, were correlated with the presence of genetic disease in NICU infants. Using a retrospective population-based cohort with extensive longitudinal follow-up, we identified clinical features that can help guide use of NGS testing in the NICU.

## Materials and Methods

This study was approved by our institutional review boards and a waiver of consent was granted due to retrospective nature of this study.

This was a retrospective cohort analysis. We identified all individuals born between January 1, 2009 and June 30, 2011 (inclusive) with a history of admission to a NICU at our system hospital, and with subsequent genetic testing (see below). Patients were then evaluated longitudinally through December 31, 2021, for any genetic testing result. Dates of presentation and length of follow-up were selected to maximize length of follow-up for up to 10 years but not go further back in time given the rapid changes in approach to genetic testing.

The health care system is a regional, not-for-profit integrated health care delivery system, with 22 hospitals, and 160 clinics and urgent care facilities located across the region, serving 60% of the state’s 3.4 million residents and 85% of the state’s children.^15^, ^16^, ^17^ The specialty clinics serve as the sole tertiary pediatric center for an estimated pediatric population of >1.7 million children,^18^ and the major site of pediatric specialty care in the region. For inclusion in the study, we used a database of genetic testing. The database contains the results of genetic panel testing, single-gene testing, Exome sequencing, and Genome sequencing that was curated by the Center for Personalized Medicine. Genetic results of the database were further cross-referenced for each patient with manual review of the electronic medical record to ensure that no results were missed. Exclusion criteria included incomplete medical records from the NICU hospitalization. A control group of patients consisted of individuals born during the same time frame but with no history of admission to the NICU. Data of the cohort were identified at different points during the analysis, and identifying participant information was necessary to link genetic testing results and patient medical records.

Data abstracted from the electronic medical record database included gestational age, birth weight, length of stay, sex, race, and ethnicity. Data reviewed and analyzed related to genetic testing included name of test, date of test, date test sample received, date test results available, and test results. Days of mechanical ventilation, inotrope administration, and antibiotic duration were obtained via manual chart review. Data pertaining to specific risk factors (ventilator days, inotrope days, and antibiotics days) were transformed into binary variables; divisions were made at 5 days for mechanical ventilation and antibiotic administration, and 3 days for vasoactive medication. These cut-offs were chosen because they were the reported average duration of these therapies in a NICU population.^19^, ^20^, ^21^, ^22^ Genetic test results were transformed into categorical variables: negative results and variant of uncertain significance were grouped together as a “negative” result, and positive results were in their own category.

Stata (StataCorp. 2021. Stata Statistical Software: Release 17: StataCorp LLC) was used for hypothesis testing. Chi-squared testing and Fisher exact were used to test for independence among categorical variables depending on size of population being tested. Logistical regression was performed to obtain odds ratios. For continuous variables, including birth weight, length of stay, and gestational age, linear regression was performed. Multivariate logistical regression analysis was performed to evaluate the relationship between multiple independent variables and the presence or absence of genetic disease.

## Results

During the study period, of patients with genetic testing, and a history of admission to a system NICU, 99 met inclusion criteria (Supplemental Figure 1). Demographics of the majority of the cohort were 60% male (*n* = 60); 91% White including Hispanic (*n* = 91); and 42% were premature (<37 weeks gestation) (*n* = 42); full details are presented in Supplemental Table 1.

In total, 64 (65%) patients had a negative (or variant of uncertain significance, [VUS]) result, and 35 (35%) had a positive (abnormal, pathogenic) genetic test. In a control cohort, the genetic test results for those without a history of NICU admission were 952 (63%) negative (or VUS) and 557 (37%) positive. The percentages of positive and negative tests between those with and without a history of NICU admission were not significantly different. The average time to a genetic test result was shorter in those who had been admitted to the NICU compared with those who had not (174.76 weeks vs 223.18 weeks *P* value < .05). The average birthweight was 2.39 kg (positive genetic test 2.14 kg vs negative test 2.54 kg *P =* .050), average gestational age was 34.9 weeks (positive genetic test 33.6 weeks vs negative test 35.6 weeks *P =* .002), and the average length of stay was 4.19 weeks (positive genetic test 5.66 weeks vs negative test 3.39 weeks *P =* .050) (Table 1).

**Table 1:** Genetic test results for patient cohort, by patient characteristic

Characteristic (N)	Genetic test result		*P* value
	Negative/VUS (*N* = 64)	Positive (*N* = 35)	
Male (60)	42 (65.6%)	18 (51%)	.2
White (94)	61 (95%)	33 (94%)	1
White Hispanic (18)	8 (12.5%)	10 (28.6%)	.06
Birth weight kg (average 2.39)	2.52 kg	2.14	.05
>5 ventilator days (12)	5	7	.08
> 5 antibiotic days (27)	16	11	.49
> 3 days of pressors (3)	1	2	.29
Gestational age, weeks (average 34.9)	35.6	33.6	.002
Average length of stay, weeks (average 4.2)	3.4	5.7	.05
Average number of risk factors (0.46)	0.34	0.57	.15
Number of risk factors			
0 risk factors	46	24	
≥ 1 risk factor	18	11	.37
≥ 2 risk factors	4	9	.05

Risk factors include >5 ventilator days, >5 antibiotic days, >3 days of pressors.

The average number of risk factors, defined as >5 ventilator days, >5 antibiotic days, or >3 days of pressors, was 0.42. On average, there was no difference in the average number of risk factors (positive genetic test, 0.57; negative/VUS test 0.34; *P =* .15). For each individual risk factor, there was no significant between the test result groups in their frequency (Table 2).

**Table 2:** Results of logistical regression of selected severity of illness indicators

Risk Factor	Odds Ratio	95% CI	*P* value
>5 ventilator days	2.95	0.86-10.12	.08
>5 antibiotic days	1.38	0.55-3.42	.49
>3 pressor days	3.82	0.33-43.68	.25
Length of stay	1.14	1.02-1.28	.02
Gestational age	0.88	0.79-0.99	.02
>2 risk factors	5.08	1.22- 21.12	.02

Length of stay and gestational age showed statistically significant differences.

We used logistical regression analysis to evaluate the odds ratio of having a positive genetic test in the presence of different clinical characteristics, as well as for each risk factor (Table 1). None of the odds ratios reached statistical significance for the risk factors. Having greater than 5 days of mechanical ventilation had an OR of 2.95 (*P =* .08). Length of stay and gestational age were both found to be statistically significant: younger gestational age had an inverse relationship with a positive test result (OR = 0.88, 95% CI = 0.79-0.99, *P =* .03); increasing length of stay had a direct correlation with positive test result (OR = 1.14, 95% CI= 1.02-1.28, *P =* .02). Multivariate logistical regression was used to evaluate the relation between the 3 variables that achieved or nearly achieved statistical significance, including birthweight, length of stay, and >5 days on the ventilator (Table 2). This multivariate analysis did not show statistical significance. However, patients who had 2 or more risk factors were roughly 5 times more likely to have a positive genetic test compared with those who did not (OR 5.08; 95% C.I. 1.22- 21.12; *P* = .02).

## Discussion

Our work suggests that clinical predictors can be used to help guide genetic testing in NICU infants. Infants with 2 or more risk factors for critical illness, indicated by longer than average duration of mechanical ventilation, pressors, or antibiotics, were more than 5 times more likely to have a positive result.^15^, ^16^, ^17^ Of the 10 patients who met these criteria, 70% had a genetic diagnosis. Previous work on genetic testing in those children with severe illness or unknown diagnosis has shown diagnostic yields from 19% to 83% with a reported change in clinical management in 7% to 60% of those children with a genetic diagnosis.^18^ Given a potential 70% diagnostic rate, the use of these clinical risk factors could provide an important diagnostic care pathway. Our study ascertainment was strengthened by use of a population-based cohort of infants, with no less than 10 years of follow-up, at a central children’s hospital that is the only provider of genetic testing and care in 500 mile radius.

An important advantage of identifying candidates for genetic testing is shortening the diagnostic odyssey and reduction in potential morbidities. The average time to genetic testing in those patients with a history of NICU admission was shorter than those without a history of NICU admission. Our study shows that it may be possible to identify candidates for genetic testing before discharge from the NICU, thus reducing the time to diagnosis given that the indicators of severity of illness utilized in this study could be identified as early as day of life 5.

Currently, most studies examining the use of genetic testing in the NICU rely on the presence of dysmorphic features, clinician judgement, unexpected response to therapy, or severe illness to identify those infants who are candidates for genetic testing.^2^^,^^4^^,^^7^^,^^10^, ^11^, ^12^, ^13^, ^14^ This study found 3 clinical features that can be easily identified and when combined with clinician judgement or unexpected response to therapy may provide an objective criteria when deciding which infants should have genetic testing.

A major advantage to our work was its extensive longitudinal follow-up, up to nearly 13 years for some patients, in a population-based cohort. Furthermore, by use of the longitudinal follow-up, we were able to identify patients who may not have been initially tested in the NICU but because of health or development concerns had subsequent genetic testing. Because the hospital and pediatric specialty groups provide nearly all of the specialized pediatric care and genetic testing in the region, the opportunity for high capture and ascertainment rates was engendered. An important consideration is that our study methodology only included genetic testing after the NICU hospitalization. Thus, we did not include infants who were diagnosed through newborn screening while in the NICU with confirmatory genetic testing, and we did not include infants who received standard of care genetic testing during NICU hospitalization. Thus, the overall proportion of infants with a positive genetic test result is higher, although in our analysis here they would be grouped in the “negative” test result denominator.

Limitations of our study includes its retrospective nature and limited sample size. The smaller sample size only included a subset of total births and only a subset of NICU admissions. Use of binary variables to assess for increased severity of illness reduced the complexity of clinical data, and in future studies, assessing these factors as continuous variables may provide more insights. Overall yield of the testing was limited by the technology available at the time, the majority of which were chromosomal microarrays and panels. This included only 5% of exome testing, no GS, and 95% panels and microarrays. Generalizability of our findings may be limited because our testing and results were only on infants and children who were referred for genetic testing. Whether the same findings would be found for unselected NICU infants is not certain. Although we found an almost 5-fold risk of a genetic diagnosis if they had 2 or more markers of critical illness, only 10% of patients met those criteria, which limits broad use. Furthermore, in the absence of defined specific phenotypes, broad genetic testing may not identify a diagnosis. Our results may have also been affected by the demographics of the study cohort, which was largely white, and has more thorough genetic variant analyses in ClinVar and other databases used for genetic pathogenicity determination. Finally, we grouped patients with a variant of uncertain significance result with those who had a negative test result, which may have biased interpretation of results. We chose to do this so as not to overestimate our findings. Some of these VUS results may be classified as pathogenic as understanding of genetic disease increases. This choice may have obscured some of the difference between these 2 groups because there are potentially patients with genetic disease that were added to the negative group.

In summary, our longitudinal, retrospective population-based study showed a correlation between clinical characteristics and the presence of pathogenic genetic test results. The rapid advances in GS technologies, including lower costs and faster turnaround for results also raises new issues whether targeted testing, as we evaluated here, versus testing all infants in the NICU, or even use of GS in newborn screening,^23^ may be considerations within the next decade. Future studies can evaluate for additional refinement of clinical characteristics, including through use of a prospective cohort.

## Conflict of Interest

The authors declare no conflicts of interest.

## Figures and Tables

**Supplementary Figure 1:**