# Supplementary material for: Clinical risk factors predicting likelihood of pathogenic genetic result in NICU patients
Source: Genet Med Open. 2026 Jan 30;4:104370. doi: 10.1016/j.gimo.2026.104370 (PMC12969806; doi:10.1016/j.gimo.2026.104370)
Supplement: Supplementary Table 1 [file mmc1.docx]

**Supplemental Figure 1.** Cohort identification. Potential participants were identified from a database of genetic test results; 2284 patients were excluded based on the study dates; 1479 patients were not included because they had no history of NICU admission; and 33 patients were not included because they had missing records. The final cohort for study was 99 patients.


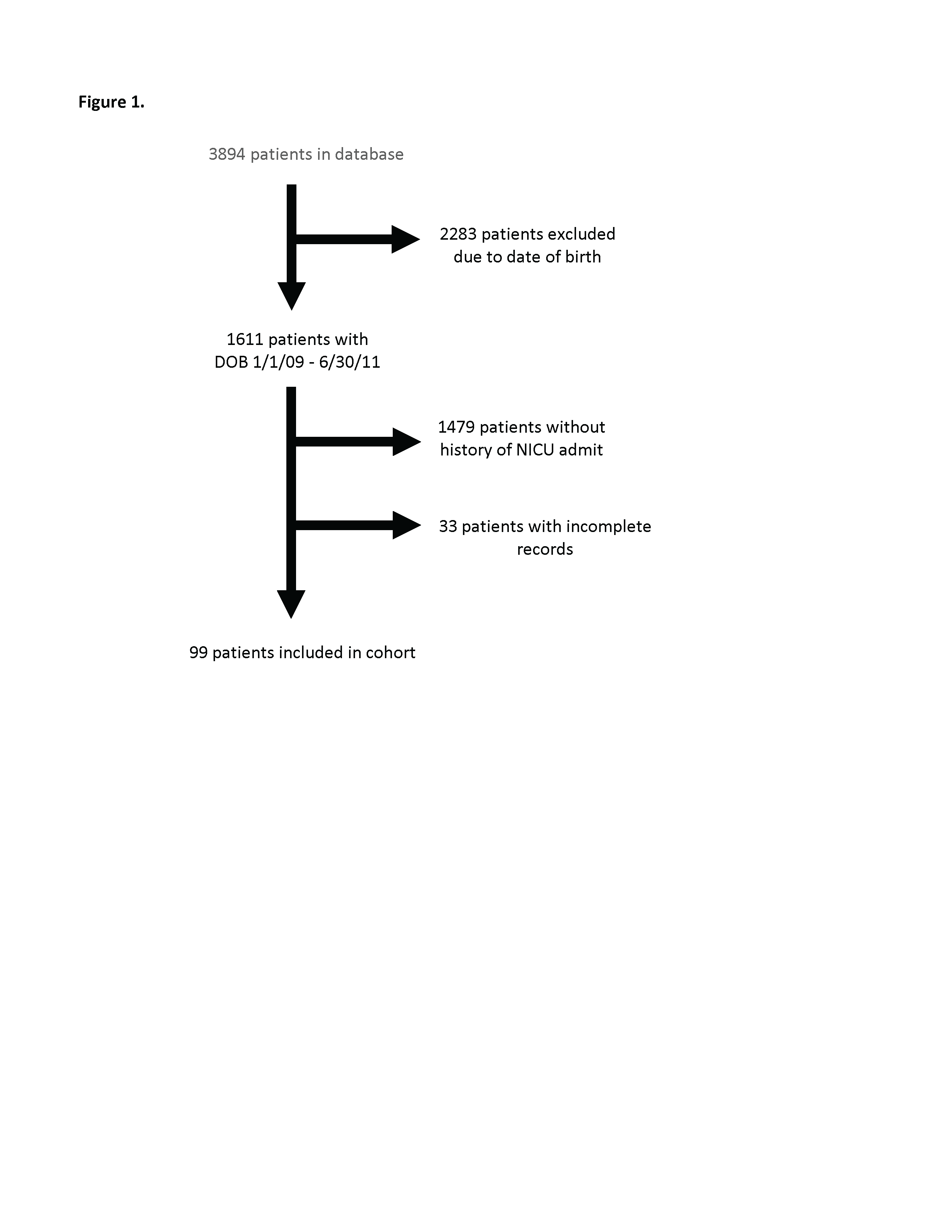


**Supplemental Table 1.** Selected demographics of the study cohort (n = 99), patients born 1/1/2009 – 06/30/2011 with both genetic testing and an IH NICU admission.

| Characteristic | Value N (%) |
| --- | --- |
| Sex |  |
| Female | 39 (39%) |
| Male | 60 (60%) |
| Race |  |
| White (Including Hispanic) | 91 (91%) |
| Other | 5 (5%) |
| Black | 2 (2%) |
| Asian/Pacific Islander | 1 (1%) |
| Premature (< 37 weeks gestation ) | 42 (42%) |
| Genetic Test Result |  |
| Positive | 35 (35%) |
| Negative or VUS | 64 (65%) |
